# Preliminary Study on Hepatocyte-Targeted Phosphorus-31 MRS Using ATP-Loaded Galactosylated Chitosan Oligosaccharide Nanoparticles

**DOI:** 10.1155/2013/512483

**Published:** 2013-12-02

**Authors:** Ri-Sheng Yu, Xiu-Liang Zhu, Jian-Zhong Sun, Dan Shi, Ying Chen, Zhi-Kang Wang, Ke-Zhong Tang, Yong-Zhong Du

**Affiliations:** ^1^Department of Radiology, The Second Affiliated Hospital, Zhejiang University School of Medicine, No. 88 Jiefang Road, Hangzhou 310009, China; ^2^Department of Pharmaceutics, College of Pharmaceutical Sciences, Zhejiang University, No. 866 Yuhangtang Road, Hangzhou 310058, China

## Abstract

*Background*. The clinical applications of hepatic phosphorus-31 magnetic resonance spectroscopy (31P MRS) remain to be difficult because the changes of phosphates between normal hepatic tissues and pathological tissues are not so obvious, and furthermore, up to now there is few literature on hepatocyte-targeted 31P MRS. *Materials and Methods*. The ATP-loaded Gal-CSO (Gal-CSO/ATP) nanoparticles were prepared and the special cellular uptake of them as evaluated by using HepG-2 tumor cells and A549 tumor cells, respectively. Two kinds of cells were incubated with the nanoparticles suspension, respectively. Then were prepared the cell samples and the enhancement efficiency of ATP peaks detected by 31P MRS was evaluated. *Results*. The cellular uptake rate of Gal-CSO/ATP nanoparticles in HepG-2 cells was higher than that in A549 cells. Furthermore, the enlarged ATP peaks of Gal-CSO/ATP nanoparticles in HepG-2 cells were higher than those in A549 cells *in vitro* detected by 31P MRS. *Conclusions*. Gal-CSO/ATP nanoparticles have significant targeting efficiency in hepatic cells *in vitro* and enhancement efficiency of ATP peaks in HepG-2 cells. Furthermore, 31P MRS could be applied in the research of hepatic molecular imaging.

## 1. Introduction

Recent progress of magnetic resonance spectroscopy (MRS) technology has great potential in many biomedical research areas. Due to the ubiquity of phosphorus-containing moieties in energy metabolism, phosphorus-31 MRS (31P MRS) has been utilized to assess energy states in living systems [1]. This technique permits simultaneous detection and quantitation of several cytosolic phosphorus-containing compounds involved in energy metabolism (adenosine triphosphate (ATP), including *γ*, *α*, and *β* signals resolved sequentially, and inorganic phosphate (Pi)) and membrane phospholipid metabolism (phosphomonoesters (PME) and phosphodiesters (PDE)) [2, 3]. These important phosphorus-containing molecules are intricately involved in the cellular processes linked to cellular destruction, turnover, and malignant transformation.

Although it holds promise as a noninvasive means of documenting the extent and progression of liver disease [4, 5] and it has been used to improve the level of diagnosis and treatment response in patients with cancer [6], the clinical applications of hepatic 31P MRS remain to be difficult because there was no significant difference of the content of phosphorus compounds between normal hepatic tissue and hepatopathy tissue *in vivo*; the 31P MRS was only used *in vitro *[3, 5]. Furthermore, to our knowledge, there are no published results on hepatocyte-targeted 31P MRS.

In recent years, nanoparticles have been extensively used to deliver drugs, genes, diagnostics, and vaccines into specific cells or tissues [7–10]. Chitosan is a naturally occurring polysaccharide obtained by deacetylation of chitin, which is a polycationic polymer comprised of mainly glucosamine units [11]. It has good biocompatibility, biodegradability, and low toxicity. The water-soluble chitosan with lower molecular weight, chitosan oligosaccharide (CSO), was obtained by enzymatic degradation in our lab [12], and like some other polycations it is known to interact with ATP by electrostatic forces of attraction to form CSO/ATP nanoparticles.

Mammalian hepatocytes possess large numbers of high-affinity, cell-surface receptors (asialoglycoprotein receptor, ASGP-R) that can bind asialoglycoproteins [13–15]. It can specifically recognize ligands with terminal galactose residues. Once a ligand binds to the ASGP-R, the ligand-receptor complex is rapidly internalized by hepatocytes, and the receptor recycled back to the surface of hepatocytes and is reutilized [15, 16], allowing high binding capacity and efficient uptake of galactosylated ligands by hepatocytes. Taking into account, we attempted to integrate a novel hepatocyte targeting carrier with multiple galactose residues.

The main goal of this study was to examine the Gal-CSO/ATP nanoparticles for hepatocyte-targeted imaging and to evaluate their targeting efficiency and the enhancement efficiency of ATP peaks detected by 31P MRS.

## 2. Materials and Methods

### 2.1. Preparation of Gal-CSO/ATP Nanoparticles

Nanoparticles were prepared successfully in our earlier research [17]. Briefly, 0.01 g Gal-CSO and 0.01 g ATP were first dissolved in 10 mL deionized water, respectively, and the mixture was stirred for 10 min by magnetic stirrer (400 rpm). Subsequently, ATP solution was dropwise mingled with the stirred Gal-CSO solution. When the transparency of the solution decreased accompanying an apparent Tyndall effect, this meant that the nanoparticles were obtained.

### 2.2. Cell Culture

Two cell lines were investigated in this study, and they are commercially available from Institute of Biochemistry and Cell Biology (Shanghai, China). HepG-2 (human hepatocellular carcinoma cell line) cells and A549 (human lung carcinoma cell line) cells were incubated in Dulbecco's Modified Eagle's Medium low glucose (DMEM), supplemented with 10% fetal bovine serum (FBS) under generally established cell culture conditions in 5% CO_2_ at 37°C. HepG-2 and A549 cells were seeded in a 24-well culture plate (Nalge Nunc International, Naperville, IL, USA), respectively, at a density of 50,000 cells per well and incubated for 24 h.

### 2.3. Nanoparticle Labeling

Synthesis of the FITC-labeled chitosan was based on the reaction between the isothiocyanate group of FITC and the primary amino group of the D-glucosamine residue as reported in the literature [18]. Briefly, 2 mg of FITC was dissolved in 1 mL of dehydrated alcohol. 0.5 mL of Gal-CSO/ATP nanoparticles were dispersed in 2 mL distilled water, respectively. After then, they were treated by ultrasonication for 20 circulations (400 W, working for 2 s following stopping for 3 s). Two of them were mixed with 100 *μ*L of FITC-alcohol solution (2.0 mg/mL), and the reaction was performed for 6 h under magnetic stirring (400 rpm) in darkness at room temperature. The FITC-labeled nanoparticles were dehydrated with pure carbinol and freeze-dried in a dark room. The nanoparticle suspension was stored in the dark room for further use.

### 2.4. Cellular Uptake

The A549 cells were used as a control. After the HepG-2 and A549 cells were cultured in 24-well plate adherent to the flask, they were then incubated with FITC-labeled Gal-CSO/ATP nanoparticles dispersion in growth medium for 24 h, respectively. Cell nuclei were stained with Hoechst for 30 min. Following the incubation, cells were washed thrice with PBS and then fixed with fresh 4% paraformaldehyde at 4°C for 20 min. The coverslips were observed by a confocal laser scanning microscope (LSM-510 META, ZEISS, Germany).

### 2.5. Quantitative Determination of Intracellular ATP Content

Two kinds of cells were seeded in a 24-well plate, respectively, at a density of 10,000 cells per well and incubated for 24 h. After then, 20 *μ*L of Gal-CSO/ATP nanoparticles suspension was added (the actual content of ATP was 1.27 mg/mL in nanoparticles) to the cells, respectively, and then further incubated for 12 h and 24 h, respectively.

After the predetermined uptake time, removed the supernatant fluid and washed the cells thrice with PBS (pH 7.4). After trypsin digestion for one minute, HCl buffer solution (pH 1.0) was added. Cells were collected in a 5 mL PB pipe at the dedicated time, respectively. The cells were stored at −80°C for 2 h and placed at room temperature; then using freeze-thaw method was used three times. After 24 h, the intracellular ATP content was determined by ultraviolet spectrophotometry. The UV wavelength was set at 259 nm. The ATP loading efficiency was then calculated from the ATP content in the water phase (PBS) during the separation process of nanoparticles and the charged amount of ATP.

### 2.6. Gal-CSO/ATP Nanoparticles on HepG-2 Cells Targeted MRS Imaging *In Vitro*

Taking A549 cells as contrast agents, we added 0.4 mL Gal-CSO/ATP nanoparticles suspension to HepG-2 cell culture solution to obtain the cell sample for MRS detection. The methods were as follows: 30 mL cell sample was placed into the cell culture bottles, followed by being put into a plastic cup (without phosphorus compounds) containing a certain amount of distilled water, and then 2 mL, 4 mL, 6 mL, 8 mL, and 10 mL ATP solutions (10 mg/mL, Beijing Double-crane Pharmaceutical LTD. Co., China) were added into the bottles, respectively. Subsequently, they were detected by 31P MRS.

All 31P MRS examinations were performed with a 1.5 T imager (Siemens, Sonata, Germany), which is equipped with a commercial dual 1H/31P surface coil for imaging of cell samples. The basic MR images in all orientations were obtained with true fast imaging with steady precession (true FISP) sequence for the localization of voxels. 31P MR spectra were measured using a standard 2-dimensional chemical shift imaging (CSI) technique in the transverse plane with the following parameters: TR = 1000 ms, TE = 2.3 ms, matrix 8 × 8, viewing interpolation 16 × 16, field of view = 200 mm, flip angle = 90 degrees, thickness = 4 cm, and voxel volume 2.5 cm × 2.5 cm × 4 cm. Spectra were evaluated using Siemens syngo 2004B software.

## 3. Results

### 3.1. Cellular Uptake Tests

**Figure 1:**
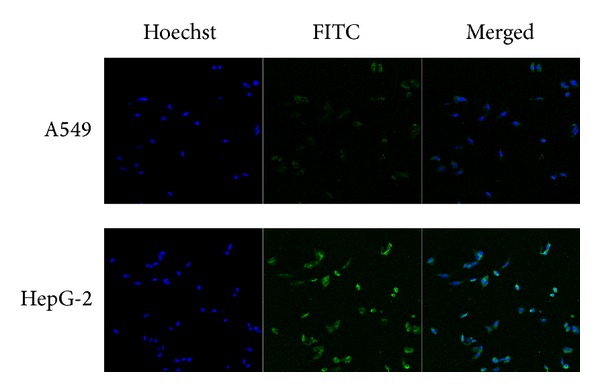
Fluorescence images of HepG-2 cells and A549 cells after the cells were incubated with FITC labeled Gal-CSO/ATP nanoparticles for 24 h, respectively. It is shown that the Gal-CSO/ATP nanoparticles could be uptaken by HepG-2 cells, and the fluorescence intensity in HepG-2 cells was stronger than that in A549 cells.

Figure 1 showed the fluorescence images of HepG-2 and A549 cells after the cells were incubated with FITC-labeled Gal-CSO/ATP nanoparticles for 24 h, respectively. It showed that the Gal-CSO/ATP nanoparticles could be uptaken by HepG-2 cells, and the fluorescence intensity in HepG-2 cells was stronger than in A549 cells. It further demonstrated that, under identical conditions, the cellular uptake capability of the Gal-CSO/ATP nanoparticles in HepG-2 cells was higher than in A549 cells.

### 3.2. Quantitative Determination of Intracellular ATP Content

**Figure 2:**
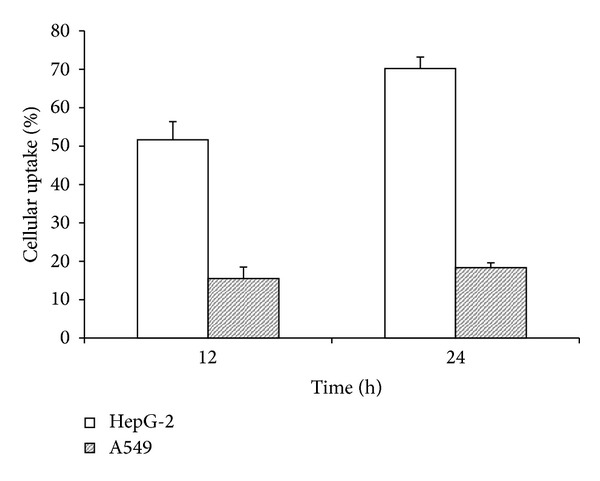
Cellular uptake percentage of Gal-CSO/ATP nanoparticles in different cell lines for 12 and 24 h, respectively. It is showen that HepG-2 cells were significantly higher than A549 cells in the cellular uptake percentage of the Gal-CSO/ATP nanoparticles.

The results of quantitative cellular uptake for FITC labeled Gal-CSO/ATP nanoparticles were presented in Figure 2 after the nanoparticles were incubated with HepG-2 and A549 cells, respectively. It was clear that HepG-2 cells were significantly higher than A549 cells in the cellular uptake percentage of the Gal-CSO/ATP nanoparticles. About 50% and 70% nanoparticles could be uptaken by HepG-2 cells in 12 and 24 h, respectively. However, the uptaken amounts of the nanoparticles by A549 cells were lower than 20% in 24 h.

### 3.3. Gal-CSO/ATP Nanoparticles on HepG-2 Cells Targeted MRS Imaging *In Vitro*

**Figure 3:**
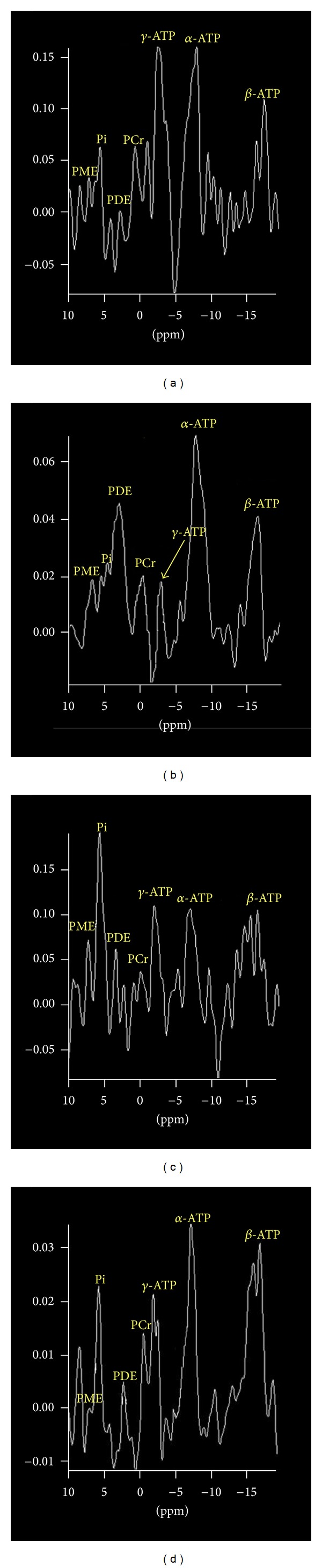
Peaks of phosphorus compounds were obtained from a solution containing HepG-2 cells. (a) MRS peaks were not ideal when adding 4 mL ATP solution; (b) MRS peaks were ideal when adding 6 mL ATP solution; (c) MRS peaks were in disorder when adding 6 mL ATP solution into the bottle containing A549 cells; and (d) MRS peaks were ideal when adding 8 mL ATP solution.

There was no significant MRS peaks of phosphorus compounds (ATP, PME, PDE and Pi) in solution containing HepG-2 cells when added into the 2 mL ATP solution mentioned above. Peaks of phosphorus compounds were detected when added into 4 mL ATP solution, but the MRS peaks were not ideal (see Figure 3(a)), while there were no peaks of phosphorus compounds detected in A549 cells solution. It was exciting that the ideal peaks of phosphorus compounds could be obtained in the solution containing HepG-2 cells by adding 6 mL ATP solution into the bottle (see Figure 3(b)), while it was quite hard for us to get peaks of phosphorus compounds in a solution containing A549 cells (see Figure 3(c)). When we added 8 mL ATP solution into the bottle, ideal peaks of phosphorus compounds emerged in solution containing HepG-2 cells, but the peaks of phosphorus compounds in the solution containing A549 cells were not so good as those of HepG-2 cells (see Figure 3(d)). And both of two bottles could get perfect MRS peaks when added 10 mL ATP to the solution.

## 4. Discussion

31P MRS imaging diagnoses the disease of liver through the peak signal intensity, which reflects the content levels of the specific phosphorylated compounds. We can achieve the purpose of MRS targeted molecular imaging by incorporation of measurable phosphide into the hepatic tissue to enlarge differences of peaks from one or more phosphorylated compounds.

One promising prospect for human hepatic 31P MRS is the measurement of hepatic energy homeostasis through the measurement of ATP, the well-known universal energy currency [19]. Previous studies have demonstrated that the measurement of hepatic ATP levels correlates with biochemical evidence of hepatic dysfunction, and histological evidence of loss of functioning hepatocytes and progressive disease, and animal models of acute liver disease [20–22]. Previous studies in our earlier research have demonstrated that Gal-CSO/ATP nanoparticles showed high encapsulation efficiency, sustained release of ATP, and efficiently delivered it to HepG-2 cells [17]. In addition, galactosylated chitosan was found to be a suitable material for liver-targeting drug/gene delivery or liver tissue engineering [23, 24], and as a hepatocyte-targeting carrier, Gal-CSO nanoparticles have a great promising potential for clinical applications due to their active liver-targeting characteristics and more than satisfactory compatibility with hepatoma cells [8]. For these reasons, in order to achieve the targeted imaging of hepatic 31P MRS and change the ATP peaks, we have synthesized novel nanoparticles, Gal-CSO/ATP nanoparticles, to improve the ATP content in the hepatic cells and to be tested as a hepatocyte-targeted carrier to evaluate its targeting efficiency and the enhancement efficiency of ATP peaks detected by 31P MRS.

In this study, Gal-CSO/ATP nanoparticles were prepared, and a fluorescent marker molecule called FITC, was encapsulated in these nanoparticles, so the qualitative study of cellular uptake of nanoparticles by HepG-2 and A549 cells could be detected by fluorescence microscope. The cell lines HepG-2 and A549 have been selected as models, because the former is well known for expressing ASGP-R [25, 26], which is present only on hepatocytes at a high density and retained on several human hepatoma cell lines, that binds and internalizes galactose-terminal (asialo)glycoprotein [27–29], and the latter one was taken as contrast agent which has no ASGP-R. The ASGP-R has been exploited as a hepatocyte-specific targeting marker for drug and gene delivery [30, 31]. To enhance the ligand-mediated endocytosis and nanoparticles uptake by the targeted cells, active targeting can be integrated with the passive targeting to enhance hepatocyte-specific delivery [32].

31P MRS findings were as follows: (1) the quantity of phosphorus compounds in solutions with different cells had no significant difference, because the similar MRS and ATP peaks were obtained from solutions with different cells added with the same ATP solution; (2) the ideal ATP peaks could be obtained by artificially increasing a certain quantity of ATP solution in the targeted cells. Furthermore, we had realized that the targeted imaging of 31P MRS by using ATP-loaded Gal-CSO nanoparticles and this work is ongoing in our lab.

## 5. Conclusions

The aforementioned results of the present study demonstrated that the Gal-CSO/ATP nanoparticles have significant targeting efficiency in liver cells *in vitro* and enhancement efficiency of ATP peaks in HepG-2 cells, and it further demonstrated that 31P MRS could be applied in the research of hepatocyte-targeted imaging. This preliminary study may be helpful to open up the field of hepatic molecular imaging and increase the clinical applications in the field of liver diseases in future.
